# Suppression Effects of Betaine-Enriched Spinach on Hyperhomocysteinemia Induced by Guanidinoacetic Acid and Choline Deficiency in Rats

**DOI:** 10.1155/2014/904501

**Published:** 2014-08-27

**Authors:** Yi-Qun Liu, Zheng Jia, Feng Han, Takahiro Inakuma, Tatsuya Miyashita, Kimio Sugiyama, Li-Cui Sun, Xue-Song Xiang, Zhen-Wu Huang

**Affiliations:** ^1^National Institute for Nutrition and Food Safety, Chinese Center for Disease Control and Prevention, 27 Nanwei Road, Xicheng District, Beijing 100050, China; ^2^Department of Clinical Medicine, Tangshan Vocational and Technical College, Tangshan 063004, China; ^3^KAGOME Co. Ltd. Research Institute, 3-14-15, Nishiki, Naka-ku, Nagoya, Aichi 460-0003, Japan; ^4^Department of Applied Biological Chemistry, Faculty of Agriculture, Shizuoka University, 836 Ohya, Suruga-ku, Shizuoka 422-8529, Japan

## Abstract

Betaine is an important natural component of rich food sources, especially spinach. Rats were fed diets with betaine or spinach powder at the same level of betaine for 10 days to investigate the dose-dependent effects of spinach powder supplementation on hyperhomocysteinemia induced by guanidinoacetic acid (GAA) addition and choline deprivation. The GAA-induced hyperhomocysteinemia in rats fed 25% casein diet (25C) was significantly suppressed by supplementation with betaine or spinach, and it was completely suppressed by taking 11.0% spinach supplementation. The choline deprivation-induced enhancement of plasma homocysteine concentration in rats fed 25% soybean protein diet (25S) was markedly suppressed by 3.82% spinach. Supplementation with betaine or spinach partially prevented the effects of GAA on hepatic concentrations of methionine metabolites. The decrease in activity of betaine-homocysteine S-methyltransferase (BHMT) and cystathionine *β*-synthase (CBS) in GAA-induced hyperhomocysteinemia was recovered by supplementation with betaine or spinach. Supplementation with betaine or spinach did not affect BHMT activity, whereas it partially restored CBS activity in choline-deprived 25S. The results indicated that betaine or spinach could completely suppress the hyperhomocysteinemia induced by choline deficiency resulting from stimulating the homocysteine removal by both remethylation and cystathionine formation.

## 1. Introduction

**Figure 1:**
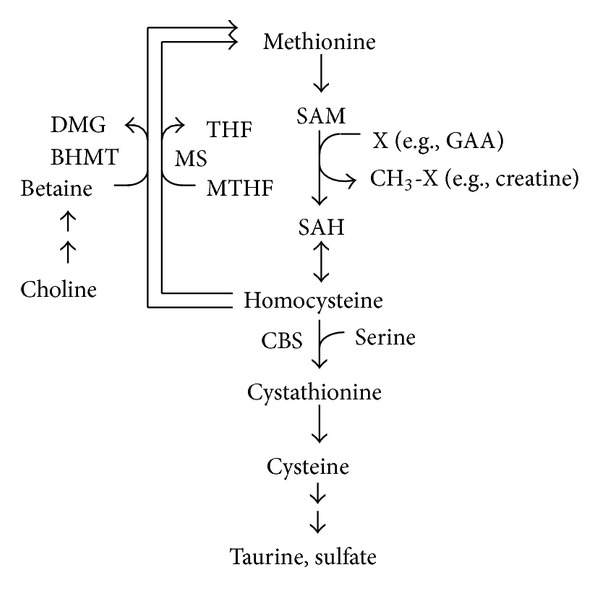
Metabolism of methionine and homocysteine.

Nutritional strategies focused on lowering plasma homocysteine concentration are beneficial to human health, because elevated plasma homocysteine levels are considered to be an independent risk factor for cardiovascular disease [1–3]. Plasma homocysteine is also a risk factor for the development of cognitive impairment and Alzheimer's disease [4]. Although it is a common amino acid, homocysteine occupies a pivotal position in the metabolism of the essential amino acid, methionine (Figure 1) [5]. It is recognized that elevated plasma homocysteine concentration causes arterial damage by several mechanisms, for example, endothelial cell injury, platelet activation, and increased oxidizability of low-density lipoproteins [3]. For human being, the normal range of plasma homocysteine concentration is 5–15 *μ*M, and 5 *μ*M increase in the concentration of this amino acid is associated with a 60–80% increased risk of coronary heart disease [2]. The plasma homocysteine concentration is affected by various factors, such as nutritional, physiological, hormonal, pharmacological, lifestyle, disease, and genetic factors [1–3]. Among these factors, genetic and nutritional factors are deemed to have greater influence on plasma homocysteine concentration. Many studies have been conducted on human to investigate the effect of dietary treatments on plasma homocysteine concentration. However, studies with experimental animals, such as rats and mice, are also useful to elucidate the regulatory mechanism of plasma homocysteine concentration. In fact, plasma homocysteine concentrations in rats are similar levels to those in human under normal conditions, and methionine loading or folate deficiency evokes hyperhomocysteinemia both in rats and in human [2].

Dietary guideline on disease prevention advises people to eat more fruits, vegetables, and grains [6]. It is commonly known that vegetables contain various micronutrients with potentially favorable effects on maintaining human health, not only by simple nutrition but also through characteristic secondary metabolites. The physiological function of vegetables and fruits has been studied throughout the world, especially in Japan [7]. Among these micronutrients, betaine is an amino acid that has received considerable interest because of its effect on human health. Recently, a number of studies were undertaken to investigate the plasma homocysteine-lowering effect of betaine on human subjects [8, 9]. The effects of betaine in various hyperhomocysteinemic animal models have also been reported, especially the models of cystathionine *β*-synthase deficiency [10], folate deficiency [11, 12], and methionine loading [13]. We have also previously reported that guanidinoacetic acid- (GAA-) induced hyperhomocysteinemia was effectively suppressed by betaine [14]. The efficacy of betaine is based on the mechanism by which the compound of hepatic betaine concentration and betaine-homocysteine S-methyltransferase (BHMT) (EC 2.1.1.5) activity [15] increase and thereby stimulates homocysteine removal by the BHMT system.

In the past few years, most interest has often been focused on folic acid supplementation because it is cheap, easy, and safe [16]. Some studies have reported that a high dietary intake of folate from vegetables and fruits decreases plasma homocysteine [17–19]. Although effects of betaine on the plasma homocysteine-lowering have been reported, which has been less thoroughly investigated on whether or not betaine from vegetables and fruits could decrease plasma homocysteine, betaine is widely found in most living organisms and it is a significant component of many foods [20, 21]. Spinach contains the highest amount of betaine among all vegetables and fruits [21–23]. In the present study, we investigated the effects of betaine and spinach powder level on hyperhomocysteinemia induced by GAA supplementation and choline deprivation.

## 2. Materials and Methods

### 2.1. Chemicals

Betaine, choline bitartrate, and GAA were purchased from Sigma-Aldrich (St. Louis, MO). Spinach powder was kindly supplied by KAGOME Co. Ltd. (Tochigi, Japan). All the other chemicals were purchased from Wako Pure Chemical Industries (Osaka, Japan) or Sigma-Aldrich and were of analytical grade. Milk casein was purchased from Nacalai Tesque (Kyoto, Japan) and soybean protein isolate (SPI) was obtained from Fuji Oil (Izumisano, Japan). Mineral mixture (AIN-93G), vitamin mixture (AIN-93), and cellulose powder were purchased from Oriental Yeast (Tokyo, Japan), and the other ingredients of the diet were purchased from Wako or Sigma-Aldrich.

### 2.2. Animals and Diets

Six-week-old male rats of the Wistar strain were obtained from Japan SLC (Hamamatsu, Japan). They were individually housed in hanging stainless-steel cages in an isolated room in which temperature (23–25°C) and humidity (40–60%) were appropriately controlled. Lighting was maintained based on a 12 h cycle (lights on from 07:00 to 19:00 h). Before starting the experiments, all rats were acclimated to the facility for 4-5 days and given free access to water and a 25% casein diet (25C), which was the same as the experimental diet described below. The experimental diets consisted of the following ingredients other than protein source (g/100 g): sucrose, 20; corn oil, 5; mineral mixture (AIN-93G), 3.5; vitamin mixture (AIN-93), 1; choline bitartrate, 0.25; cellulose, 2; and cornstarch to make 100. Alteration of types of dietary protein and addition of supplement(s) were made by adjusting the content of cornstarch. Two experiments were conducted in this study. In experiment 1, rats were fed 25C, 25C + 0.5% GAA (25CG) diets with different betaine levels (0.05, 0.1, and 0.2%) and spinach powder levels (2.76, 5.52, and 11.1%) corresponding to betaine levels for 10 days to investigate the dose-dependent effects of spinach powder supplementation on hyperhomocysteinemia induced by GAA addition. In experiment 2, rats were fed 25% soybean protein diet (25S), a choline-deficient 25S (25SCD) diets with different betaine levels (0.05 and 0.1%) and spinach powder levels (1.91 and 3.82%) at the same level of betaine for 10 days to investigate the dose-dependent effects of spinach powder supplementation on hyperhomocysteinemia induced by choline deprivation. The addition level of GAA was determined based on the results of our previous studies [24]. The experimental period of 10 days was sufficient to induce stable hyperhomocysteinemia by addition of GAA [24] or choline deprivation [25]. This study was approved by the Animal Use Committee of Shizuoka University, and the animals were maintained in accordance with the “Guidelines for the Care and Use of Laboratory Animals” of Shizuoka University.

### 2.3. Tissue Collection and Fractionation

Rats were killed by decapitation to obtain blood and livers between 10:00 and 11:00 h without prior food deprivation. Blood plasma was separated from heparinized whole blood by centrifugation at 2,000 ×g for 15 min at 4°C and stored at −30°C until needed for analysis. After blood collection, the whole liver was quickly removed, rinsed in ice-cold saline, blotted on filter paper, cut into two parts, weighed, quickly frozen in liquid nitrogen, and stored at −80°C until analysis. One part of the liver was homogenized in 4 volumes (vol/wt) of ice-cold 0.3 M trichloroacetic acid solution and then centrifuged at 10,000 ×g for 10 min at 4°C. The supernatant of the deproteinized liver homogenate was subject to assay of methionine metabolites, betaine, and serine. The other portion of the liver was homogenized in 4 volumes (vol/wt) of 10 mM sodium phosphate buffer (pH 7.4) containing 0.15 M KCl, and the resulting homogenate was centrifuged at 14,000 ×g for 10 min at 4°C. The supernatant was subject to enzyme assays.

### 2.4. Biochemical Analysis

The concentrations of total homocysteine and cysteine in plasma and liver were measured by HPLC according to the method of Durand et al. [26]. The methods of Cook et al. [27] and Laryea et al. [28] were employed to determine the concentrations of S-adenosylmethionine (SAM) and S-adenosylhomocysteine (SAH) as well as betaine in the liver by using the HPLC system, respectively. The concentration of serine in the liver was measured by an amino acid autoanalyzer (Model L-8500; Hitachi). The activity of CBS in the liver was determined according to the method of Mudd et al. [29], but HPLC was used in the assay of the reaction product, cystathionine, described by Einarsson et al. [30]. The activity of BHMT in the liver was measured according to the method of Finkelstein and Mudd [31], but HPLC was used in the assay of the reaction product, DMG, described by Laryea et al. The protein concentration in liver was measured by the method of Lowry et al. [32] using bovine serum albumin as a standard.

### 2.5. Statistical Analysis

Data are expressed as means ± square estimated margin (SEM). Data were analyzed by a one-way ANOVA, and the differences among groups were tested by the Tukey test when the *F* value was significant. The statistical analysis was performed using Mac Toukei-Kaiseki ver 1.5 software (Esumi, Tokyo, Japan).

## 3. Results

### 3.1. Effect on Guanidinoacetic Acid-Induced Hyperhomocysteinemia (Experiment 1)

**Figure 2:**
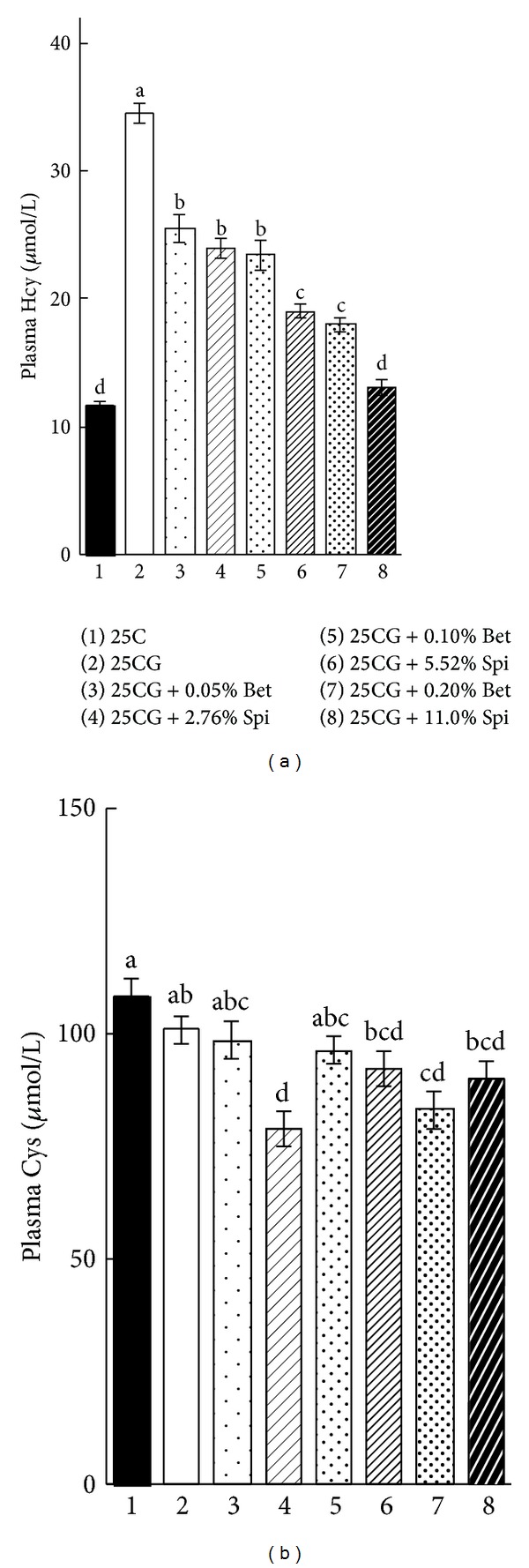
Plasma homocysteine (a) and cysteine (b) concentrations in rats fed the experimental diets (experiment 1). Each value is the mean ± SEM, *n* = 8. Values in a panel without a common letter differ at *P* < 0.05. 25C, 25% casein diet; 25CG, 25C + 0.5% guanidinoacetic acid; Bet, betaine; Cys, cysteine; Hcy, homocysteine; Spi, spinach powder.

Body weight gain, food intake, and relative liver weight were not affected by the addition of GAA and supplementation with betaine or spinach powder level (Table 1), except that the relative liver weight was slightly lower in those rats fed with 0.1% betaine or 5.52% spinach powder diet than the rats compared to the other diets. The addition of GAA significantly increased plasma homocysteine concentration from 11.8 ± 0.3 (25C group) to 34.5 ± 0.8 *μ*mol/L (25CG group) (Figure 2(a)). The GAA-induced hyperhomocysteinemia was significantly suppressed by supplementation with betaine or spinach powder in a dose-dependent manner, and it was completely suppressed by 11.0% of spinach powder supplementation. Supplementation with 0.2% betaine or spinach powder significantly decreased or tended to decrease the plasma cysteine concentration (Figure 2(b)).

**Figure 3:**
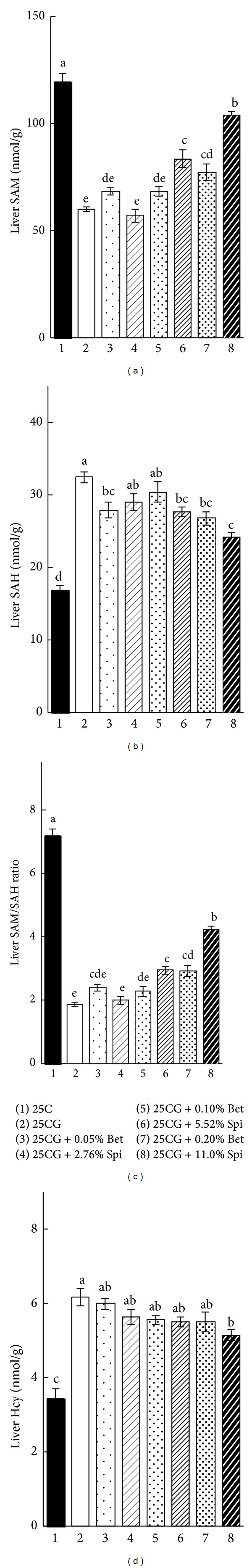
Hepatic concentrations of S-adenosylmethionine (a) and S-adenosylhomocysteine (b), their ratio (c), and homocysteine (d) in rats fed the experimental diets (experiment 1). Each value is the mean ± SEM, *n* = 8. Values in a panel without a common letter differ at *P* < 0.05.

The addition of GAA significantly reduced hepatic SAM concentration and SAM/SAH ratio; on the contrary, it raised hepatic SAH and homocysteine concentrations (Figures 3(a)–3(d)). Supplementation with betaine or spinach powder partially prevented the effects of GAA on hepatic concentrations of methionine metabolites. Among the experimental groups, however, there was significant effect on supplementation with 0.2% betaine or spinach powder (5.52% and 11.0%). And the hepatic homocysteine concentration was significantly lower in the rats fed diets supplemented with 11.0% spinach powder than in those fed with the other diets.

**Figure 4:**
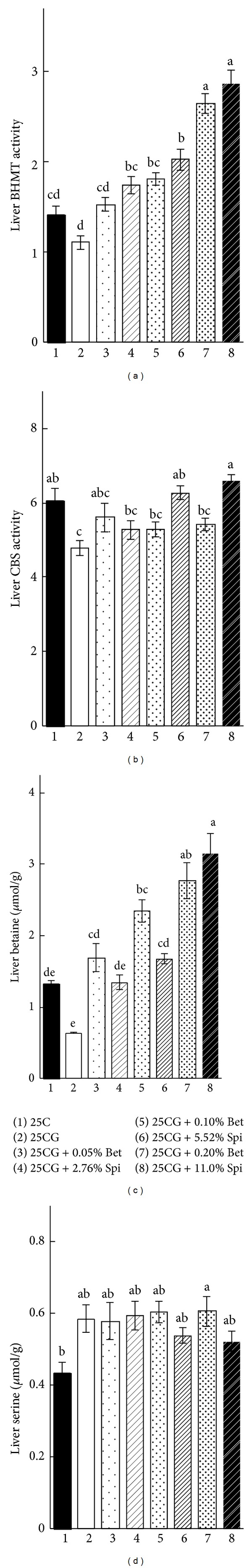
Activities of betaine-homocysteine S-methyltransferase (a) and cystathionine *β*-synthase (b) and concentrations of  betaine (c) and serine (d) in the liver of rats fed the experimental diets (experiment 1). Each value is the mean ± SEM, *n* = 8. Values in a panel without a common letter differ at *P* < 0.05. Enzyme activities are expressed in nmol/(min*·*mg protein).

The addition of GAA decreased the hepatic betaine concentration, together with hepatic BHMT and CBS activities, whereas it slightly increased the hepatic serine concentration (Figures 4(a)–4(d)). The decrease in betaine concentration and BHMT activity were completely restored and further increased to the level even higher than those rats fed 25C with supplementation with 0.20% betaine or 11.0% spinach powder. The decrease in CBS activity was restored by supplementation with betaine or spinach powder in a dose-dependent fashion. In contrast, supplementation with betaine or spinach powder did not affect the serine concentration.

### 3.2. Effect on Choline Deprivation-Induced Hyperhomocysteinemia (Experiment 2)

**Figure 5:**
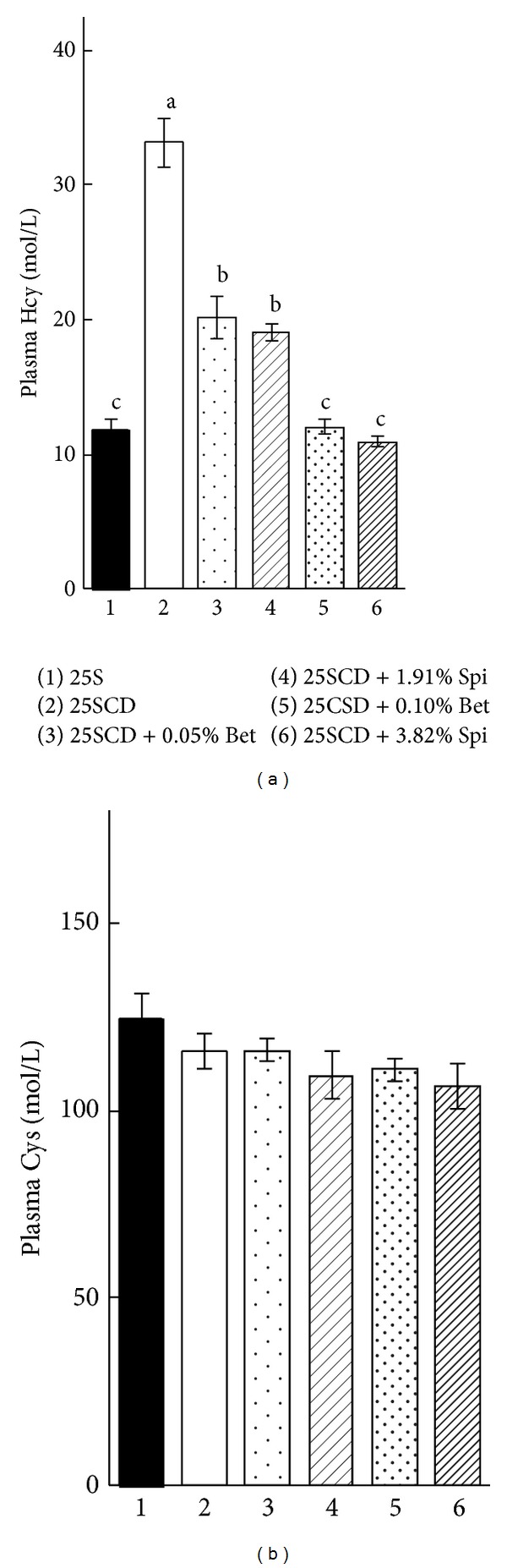
Plasma homocysteine (a) and cysteine (b) concentrations in rats fed the experimental diets (experiment 2). Each value is the mean ± SEM, *n* = 6. Values in a panel without a common letter differ at *P* < 0.05. 25S, 25% soybean protein diet; 25SCD, choline-deprived 25S. See the caption of Figure 2 for other abbreviations.

Body weight gain and food intake made no difference among the experimental groups (Table 1), whereas the relative liver weight was slightly higher in rats fed with the dietary of choline deprivation and was slightly lower in rats fed with 0.1% betaine diet compared to the other diets. Choline deprivation of 25S significantly increased plasma homocysteine concentration from 11.8 ± 0.7 (25S group) to 33.2 ± 1.8 *μ*mol/L (25SCD group) (Figure 5(a)). The choline deprivation-induced enhancement of plasma homocysteine concentration was significantly suppressed by betaine or spinach powder supplementation in a dose-dependent manner. Plasma cysteine concentration had no significant difference among the experimental groups (Figure 5(b)).

**Figure 6:**
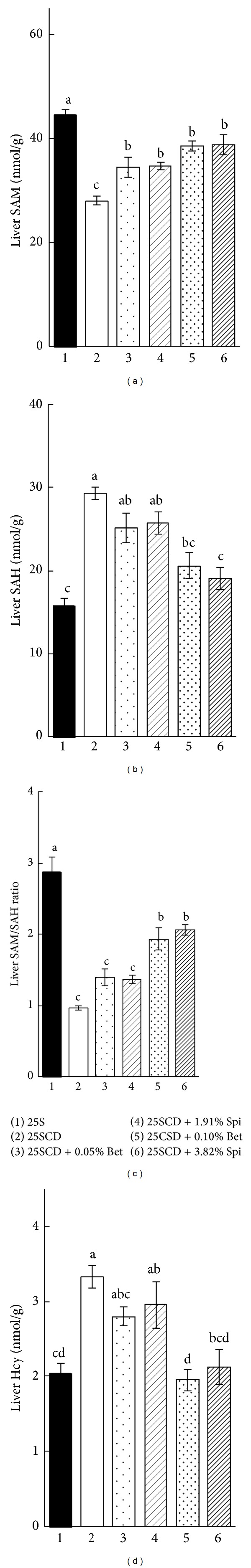
Hepatic concentrations of S-adenosylmethionine (a) and S-adenosylhomocysteine (b), their ratio (c), and homocysteine (d) in rats fed the experimental diets (experiment 2). Each value is the mean ± SEM, *n* = 6. Values in a panel without a common letter differ at *P* < 0.05.

Choline deprivation significantly decreased hepatic SAM concentration and SAM/SAH ratio and, conversely, increased hepatic SAH and homocysteine concentrations (Figures 6(a)–6(d)). The choline deprivation-induced decrease in hepatic SAM concentration was suppressed by betaine or spinach powder supplementation. The increase in hepatic SAH concentration had a dose-dependent suppression by betaine or spinach powder supplementation. The decrease in SAM : SAH ratio was significantly suppressed by 0.1% betaine or 3.82% spinach powder supplementation. The increase in hepatic homocysteine concentration was significantly suppressed by betaine or spinach powder supplementation. The profile of hepatic homocysteine concentration was similar to that of plasma homocysteine concentration.

**Figure 7:**
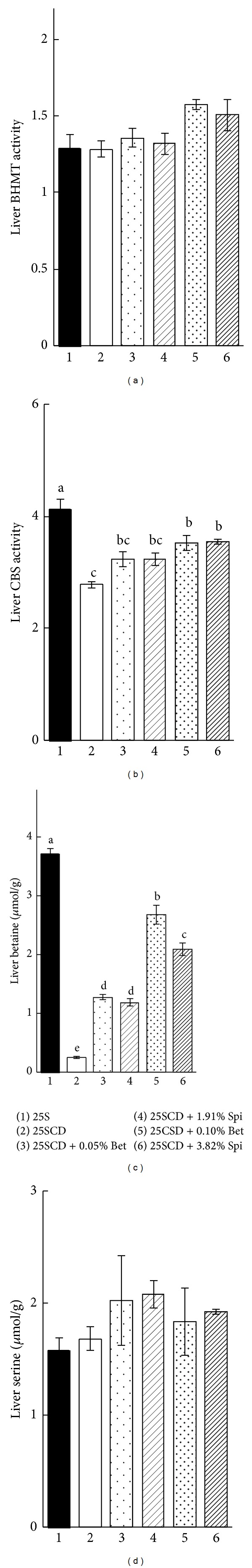
Activities of betaine-homocysteine S-methyltransferase (a) and cystathionine *β*-synthase (b) and concentrations of betaine (c) and serine (d) in the liver of rats fed the experimental diets (experiment 2). Each value is the mean ± SEM, *n* = 6. Values in a panel without a common letter differ at *P* < 0.05. Enzyme activities are expressed in nmol/(min*·*mg protein).

Choline deprivation significantly decreased hepatic betaine concentration from 3.72 ± 0.09 (25S group) to 0.25 ± 0.02 *μ*mol/g (25SCD group), together with hepatic CBS activity, whereas it did not affect hepatic BHMT activity and serine concentration (Figures 7(a)–7(d)). Supplementation with betaine or spinach powder did not affect hepatic BHMT activity and serine concentration, whereas it partially restored hepatic CBS activity. The decrease in hepatic betaine concentration was partially dose dependently suppressed by betaine or spinach powder supplementation.

## 4. Discussion

Several studies have suggested that dietary betaine level might be a determinant of plasma homocysteine concentration [10–12]. It is usually assumed that dietary betaine affects homocysteine metabolism by stimulating homocysteine remethylation; a change in BHMT activity is essential to the hyperhomocysteinemic effects of choline and betaine, since a study by Finkelstein et al. [15] showed that dietary supplementation with choline or betaine at a level of 0.2% increased hepatic BHMT activity in rats fed low-methionine diets without choline. Moreover, other mechanisms have also been considered. For instance, the restoration of the hepatic SAM concentration can be associated with the effect of choline or betaine under some physiological conditions, since the SAM concentration affects the activity of CBS in an allosteric manner [33]. Indeed, a decreased SAM concentration was one of the features of hyperhomocysteinemia induced by folate deficiency [34] and by GAA supplementation [24]. The present study was conducted to clarify these points by using experimental hyperhomocysteinemic rats and assess the effect of dietary betaine and spinach powder. In this study, the average plasma homocysteine concentration decreased at most by 62% or 66% in response to the diet of high level of supplementation with spinach powder in hyperhomocysteinemia induced by the addition of GAA or choline deficiency, respectively. The results obtained here clearly demonstrated that spinach completely suppressed hyperhomocysteinemia induced by the above-mentioned models. Also interesting findings of experiment 1 were that the suppressive effect of betaine was not stronger than spinach compared with the same content of betaine, although betaine effectively suppressed the hyperhomocysteinemia.

GAA is a precursor of creatine which is phosphorylated and plays an essential role as a high-energy carrier in muscle. It has been estimated that about 75% of labile methyl groups is used to synthesize creatine by a reaction catalyzed by GAA N-methyltransferase [35, 36]. Stead et al. [37] have first provided evidence that dietary supplementation with 0.34% GAA increased the plasma homocysteine concentration in rats, and they inferred that GAA supplementation increased the plasma homocysteine concentration by accelerating the conversion of SAM to SAH and further to homocysteine. Based on this theory, we reported that supplementation with GAA led to the decrease in the hepatic SAM concentration and increase in the hepatic SAH and homocysteine concentration in a dose-dependent manner in rats [24]. In addition, we previously assumed that betaine deficiency might contribute to GAA-induced hyperhomocysteinemia, based on the finding that GAA supplementation significantly decreased the hepatic betaine concentration [14, 38]. There are several possible mechanisms for the GAA-induced decrease in hepatic betaine concentration: (i) increased consumption of betaine due to acceleration of the methionine cycle by GAA loading, (ii) decreased synthesis of PC via the PE N-methylation pathway due to a decrease in hepatic SAM concentration, inhibition by increased SAH concentration, or both, and (iii) decreased synthesis of PC via the PE N-methylation pathway due to competition between PE N-methyltransferase and GAA N-methyltransferase for SAM. The present study indicated that hyperhomocysteinemia could be partially suppressed by the addition of betaine, whereas it could be completely suppressed by supplementation with spinach which was comparable to the same content of betaine. One possible explanation for the reasons might be due to folate content of the dietary spinach. Although 5-methyltetrahydrofolate can donate its methyl group to homocysteine, this pathway might not be as functional as the betaine pathway, because folate did not suppress GAA-induced hyperhomocysteinemia when added to the diet at up to 40 mg/kg (unpublished data). Hence, another possible explanation is due to choline, since its concentration in spinach is much higher comparing with other vegetables and fruits [21]. It appears that choline restored PC synthesis through the latter two mechanisms, since the facts that spinach supplementation significantly increased hepatic SAM concentration while decreasing hepatic SAH concentration are favorable to the restoration of decreased PC synthesis via the PE N-methylation pathway. Consistent with our previous study [14], this result also suggested that the addition of spinach exerts betaine and choline effects by increasing both the hepatic betaine concentration and BHMT activity significantly. Furthermore, an increase in hepatic CBS activity might also contribute to the plasma homocysteine-normalizing effects of betaine and choline.

As it is well known, betaine and choline are lipotropic factors that stimulate PC synthesis and prevent the development of fatty liver [39, 40]. In addition, most studies have reported that betaine administration was effective in decreasing plasma homocysteine concentration [8, 12]. We previously showed that choline deprivation-induced hyperhomocysteinemia could be effectively suppressed by two kinds of lipotrope [25]. The results of the present study clearly showed that supplementation with betaine or spinach completely suppressed the hyperhomocysteinemia induced by choline deficiency.

Varela-Moreiras et al. [41] first demonstrated that choline deprivation in the diet, which contained methionine at a level of 0.2%, increased serum homocysteine concentration. We also showed that choline deprivation gave rise to hyperhomocysteinemia in rats fed with a 10% casein diet or 25% soybean protein diet (25S), whereas it did not elevate plasma homocysteine concentration in rats fed with a 25% casein diet [25]. It is proved that choline deprivation-induced hyperhomocysteinemia is associated with choline deficiency, since it was accompanied by the development of fatty liver, which is one of the indices of deficiency of phosphatidylcholine (PC) and choline [42]. PC is synthesized by two pathways, the CDP-choline pathway and phosphatidylethanolamine (PE) N-methylation pathway [43, 44]. The results of the present study showing that hyperhomocysteinemia induced by choline deprivation increases in hepatic SAH and homocysteine concentrations suggest that suppressed removal of homocysteine was associated with the hyperhomocysteinemia. Consequently, at least two possible mechanisms are considered for choline deficiency-induced hyperhomocysteinemia: (i) suppressed remethylation of homocysteine due to a decrease in hepatic betaine concentration and (ii) decreased formation of cystathionine due to a decrease in hepatic SAM concentration. It has been shown that the hepatic BHMT activity was influenced by some dietary conditions, for example, choline, betaine, and methionine [15, 45]. In addition to the enzyme activity, the availability of betaine is also considered to affect the remethylation reaction catalyzed by BHMT. In this study, the activity of BHMT was unaffected by choline deprivation. In contrast, hepatic betaine concentration was markedly decreased by choline deprivation to the level of 0.24 ± 0.02 *μ*mol/g in rats fed with the 25S diet. It has been reported that the Km value for betaine was 120 *μ*M in rat liver purified BHMT [46] and 48 *μ*M in rat liver semipurified BHMT [47]. When the data obtained are adopted, the decreased betaine concentration in rats fed with 25SCD diet appears to be lower than the saturable betaine concentration for BHMT, for example,two-fold the Km value or more. Thus, it is likely that choline deprivation decreases betaine-dependent remethylation of homocysteine through a decrease in hepatic betaine concentration rather than the alternatives of hepatic BHMT activity, at least in rats fed with the 25S diet. Our results also suggest that betaine or spinach significantly increased the hepatic betaine concentration and tended to increase BHMT activity.

In addition, choline deprivation significantly decreased both hepatic SAM concentration and CBS activity in rats fed with 25S diet in this study, since SAM concentration affected the activity of CBS [33]. Therefore, it appears that decreased CBS activity may also favor the induction of hyperhomocysteinemia by choline deficiency in rats fed with 25S diet. It has been reported, however, that choline deficiency caused secondary folate deficiency [48] and vice versa [49]. Therefore, the possibility that the decreased remethylation of homocysteine due to secondary folate deficiency is also involved in choline deficiency-induced hyperhomocysteinemia cannot be ignored.

## 5. Conclusions

The present study demonstrated that decreased CBS activity was partially restored by supplementation with betaine or spinach. The findings of this study suggested that betaine or spinach could completely suppress the hyperhomocysteinemia induced by choline deficiency due to stimulation of the homocysteine removal by both remethylation and cystathionine formation.

## Figures and Tables

**Table 1:** Body weight gain, food intake, and liver weight of rats fed the experimental diets (experiments 1 and 2)^1^.

Diet	Body weight gain g/10 d	Food intake g/10 d	Liver weight g/100 g body weight
Experiment 1			
25C	42.8 ± 1.6	126 ± 5	4.49 ± 0.04^a^
25CG	46.2 ± 2.3	125 ± 3	4.28 ± 0.07^ab^
25CG + 0.05% Bet	44.5 ± 2.2	127 ± 6	4.28 ± 0.07^ab^
25CG + 2.76% Spi	50.4 ± 2.5	122 ± 3	4.25 ± 0.05^ab^
25CG + 0.10% Bet	43.2 ± 2.2	122 ± 3	4.21 ± 0.04^b^
25CG + 5.52% Spi	49.6 ± 1.8	125 ± 4	4.17 ± 0.04^b^
25CG + 0.20% Bet	48.8 ± 2.3	128 ± 3	4.27 ± 0.06^ab^
25CG + 11.1% Spi	50.2 ± 1.5	122 ± 1	4.33 ± 0.05^ab^
Experiment 2			
25S	40.5 ± 1.5	136 ± 4	4.17 ± 0.05^ab^
25SCD	40.0 ± 1.8	140 ± 3	4.30 ± 0.05^a^
25SCD + 0.05% Bet	44.0 ± 3.0	142 ± 3	4.23 ± 0.04^ab^
25SCD + 1.91% Spi	46.9 ± 1.8	143 ± 2	4.27 ± 0.06^ab^
25SCD + 0.10% Bet	43.3 ± 3.5	135 ± 5	4.05 ± 0.05^b^
25SCD + 3.82% Spi	48.4 ± 2.0	138 ± 4	4.08 ± 0.08^ab^

^1^Each value is the mean ± SEM, *n* = 8. Values with different letters are significantly different at *P* < 0.05. 25C, 25% casein diet; 25CG, 25C + 0.5% guanidinoacetic acid; 25S, 25% soybean protein diet; 25SCD, choline-deprived 25S; Bet, betaine; Spi, spinach.
